# Complete Genome Sequence of Helicobacter pylori Strain 3192, Isolated from a Chinese Patient with Chronic Nonatrophic Gastritis

**DOI:** 10.1128/mra.00382-22

**Published:** 2022-06-02

**Authors:** Fang Jin, Hong Yang

**Affiliations:** a State Key Laboratory of Microbial Metabolism, School of Life Sciences and Biotechnology, Shanghai Jiao Tong University, Shanghai, China; University of Maryland School of Medicine

## Abstract

Here, we present the complete genome sequence of Helicobacter pylori 3192, isolated from a patient diagnosed with nonatrophic gastritis in China. The genome is 1.6 Mbp long and encompasses 1,566 genes, including *cagA* and *vacA* genes.

## ANNOUNCEMENT

Helicobacter pylori is closely related with many digestive system diseases, including chronic active gastritis, gastroduodenal ulcer, gastric mucosa-associated lymphoid tissue lymphoma, and gastric cancer (1). The H. pylori biofilm can significantly improve antibiotic resistance, which poses a challenge to the treatment of infected humans (2). Among 10 H. pylori strains, strain 3192 could form more biofilms at the gas-liquid interface in Brucella broth medium (Qingdao Hope Bio-technology Co., Ltd., China) containing 2% Gibco fetal serum (3). Here, we report the complete genome sequence of H. pylori 3192 under the World Medical Association (WMA) Declaration of Helsinki (https://www.wma.net/policies-post/wma-declaration-of-helsinki-ethical-principles-for-medical-research-involving-human-subjects/).

H. pylori 3192 was isolated from a patient with chronic nonatrophic gastritis in China and cultured on Columbia blood agar plates containing 5% sterile defibrinated sheep blood for 48 to 72 h at 37°C under microaerobic conditions. After a single colony picked from a plate grew in Brucella broth containing 2% fetal serum for 48 to 72h, its genomic DNA was extracted by a modified phenol-chloroform method (4). Genomic DNA was sequenced using the Next Ultra DNA library prep kit for Illumina (New England BioLabs [NEB], USA) on the Illumina Novaseq 6000 platform generating 150-bp paired-end reads and the SQK-LSK109 sequencing kit protocol (Oxford Nanopore Technologies, Oxford, UK) on the Nanopore PromethION platform. For Illumina sequencing, 7,181,160 raw reads (~654.9× coverage) were trimmed with sickle v1.33 (5) by removing low-quality bases at both ends of the sequence and the sequences with joints, a low average quality of bases, multiple Ns, or a short length. A total of 7,120,712 clean reads were generated. For Oxford Nanopore sequencing, GUPPY v5.0.16 (6) (https://staff.aist.go.jp/yutaka.ueno/guppy/) was used for base calling, 158,531 raw reads (~1,073.1× coverage) were quality controlled with NanoPlot v1.15.0 (7) (a threshold quality [Q] value of ≥7), and 150,516 clean reads were generated with an average length of 199,77 bp and an *N*_50_ value of 21,632 bp.

The *de novo* genome was assembled by Unicycler v0.4.9 (8) and optimized by arrow v2.3.2 (9) to generate one single circular contig that was 1,637,585 bp long and had GC content of 38.72%; the genome coverage was 604.0×. NCBI Prokaryotic Genome Annotation Pipeline (PGAP) v5.3 (10) was used to annotate the genome. It predicted a total of 1,566 genes with 1,453 protein-coding sequences, 2 complete sets of rRNAs (5S, 16S, and 23S rRNAs), 36 tRNAs, and 3 noncoding RNAs (ncRNAs). The types of *vacA* and *cagA* genes were further analyzed by PCR and MEGAX v10.1.8 (11) separately, and PCR primers and software parameters were same as those of Yamazaki’s research (12). The genome contains the major virulence factor Western-type *cagA* (3,518 bp), S1a/m2 type *vacA* (3,911 bp), *babA* (2,225 bp), and *sabA* (1,946 bp) (12, 13). Default parameters were used for all software unless otherwise specified.

### Data availability.

The sequence read files and the genome sequences of H. pylori strain 3192 have been deposited in the GenBank database with the accession number CP086760. The raw data were deposited in the Sequence Read Archive (SRA) with the accession number SRR18740288 and SRR18210285.

## References

[1] Goderska K, Pena SA, Alarcon T. 2018. *Helicobacter pylori* treatment: antibiotics or probiotics. Appl Microbiol Biotechnol 102:1–7. doi:10.1007/s00253-017-8535-7. 29075827 PMC5748437

[2] Hathroubi S, Servetas SL, Windham I, Merrell DS, Ottemann KM. 2018. *Helicobacter pylori* biofilm formation and its potential role in pathogenesis. Microbiol Mol Biol Rev 82:e00001-18. doi:10.1128/MMBR.00001-18. 29743338 PMC5968456

[3] Jin F, Yang H. 2021. Effects of *Lactobacillus salivarius* LN12 in combination with amoxicillin and clarithromycin on *Helicobacter pylori* biofilm in vitro. Microorganisms 9:1611. doi:10.3390/microorganisms9081611. 34442690 PMC8399496

[4] Wang N, Hang XM, Zhang M, Peng XY, Yang H. 2017. New genetic environments of the macrolide-lincosamide-streptogramin resistance determinant *erm(X)* and their influence on potential horizontal transferability in *bifidobacteria*. Int J Antimicrob Agents 50:572–580. doi:10.1016/j.ijantimicag.2017.04.007. 28666750

[5] Joshi NAFJ. 2011. Sickle: a sliding-window, adaptive, quality-based trimming tool for FastQ files. Version 1.33. https://github.com/najoshi/sickle.

[6] Wick RR, Judd LM, Holt KE. 2019. Performance of neural network basecalling tools for Oxford Nanopore sequencing. Genome Biol 20:129. doi:10.1186/s13059-019-1727-y. 31234903 PMC6591954

[7] De Coster W, D'Hert S, Schultz DT, Cruts M, Van Broeckhoven C. 2018. NanoPack: visualizing and processing long-read sequencing data. Bioinformatics 34:2666–2669. doi:10.1093/bioinformatics/bty149. 29547981 PMC6061794

[8] Wick RR, Judd LM, Gorrie CL, Holt KE. 2017. Unicycler: resolving bacterial genome assemblies from short and long sequencing reads. PLoS Comput Biol 13:e1005595. doi:10.1371/journal.pcbi.1005595. 28594827 PMC5481147

[9] Chin CS, Alexander DH, Marks P, Klammer AA, Drake J, Heiner C, Clum A, Copeland A, Huddleston J, Eichler EE, Turner SW, Korlach J. 2013. Nonhybrid, finished microbial genome assemblies from long-read SMRT sequencing data. Nat Methods 10:563–569. doi:10.1038/nmeth.2474. 23644548

[10] Tatusova T, DiCuccio M, Badretdin A, Chetvernin V, Nawrocki EP, Zaslavsky L, Lomsadze A, Pruitt KD, Borodovsky M, Ostell J. 2016. NCBI Prokaryotic Genome Annotation Pipeline. Nucleic Acids Res 44:6614–6624. doi:10.1093/nar/gkw569. 27342282 PMC5001611

[11] Stecher G, Tamura K, Kumar S. 2020. Molecular Evolutionary Genetics Analysis (MEGA) for macOS. Mol Biol Evol 37:1237–1239. doi:10.1093/molbev/msz312. 31904846 PMC7086165

[12] Yamazaki S, Yamakawa A, Okuda T, Ohtani M, Suto H, Ito Y, Yamazaki Y, Keida Y, Higashi H, Hatakeyama M, Azuma T. 2005. Distinct diversity of *vacA*, *cagA*, and *cagE* genes of *Helicobacter pylori* associated with peptic ulcer in Japan. J Clin Microbiol 43:3906–3916. doi:10.1128/JCM.43.8.3906-3916.2005. 16081930 PMC1233989

[13] Baj J, Forma A, Sitarz M, Portincasa P, Garruti G, Krasowska D, Maciejewski R. 2020. *Helicobacter pylori* virulence factors-mechanisms of bacterial pathogenicity in the gastric microenvironment. Cells 10:27. doi:10.3390/cells10010027. PMC7824444 33375694

